# Quantification of Trimethylamine-*N*-Oxide and Trimethylamine in Fish Oils for Human Consumption

**DOI:** 10.3390/molecules29061339

**Published:** 2024-03-17

**Authors:** Dominik Dörfel, Sascha Rohn, Eckard Jantzen

**Affiliations:** 1 GALAB Laboratories GmbH, Am Schleusengraben 7, 21029 Hamburg, Germany; eckard.jantzen@galab.de; 2 Department of Food Chemistry and Analysis, Institute of Food Technology and Food Chemistry, Technical University Berlin, Gustav-Meyer-Allee 25, 13355 Berlin, Germany; rohn@tu-berlin.de

**Keywords:** trimethylamine-*N*-oxide, trimethylamine, fish oil, HILIC-MS/MS, cardiovascular disease, liquid extraction

## Abstract

Supplementing fish oil is one of the strategies to reduce the risk of cardiovascular disease, the leading cause of death around the world. Contradictorily, fish oil may also contain trimethylamine-*N*-oxide, a recently emerged risk factor for cardiovascular disease, as well as one of its precursors, trimethylamine. A method suitable for routine quantification of trimethylamine-*N*-oxide and trimethylamine in fish oil with a quick and easy liquid extraction without derivatization has been developed. Liquid chromatography with tandem mass spectrometry detection was employed along with a zwitterionic hydrophilic interaction liquid chromatography column and a gradient elution with eluents containing 50 mmol/L of ammonium formate. An internal standard (triethylamine) was used for quantification by mass spectrometry with an external calibration. The assay proved high linearity in the ranges of 10 to 100 ng/mL and 100 to 1000 ng/mL for trimethylamine-*N*-oxide and trimethylamine, respectively. The lowest limit of quantification was determined to be 100 µg/kg for trimethylamine and 10 µg/kg for trimethylamine-*N*-oxide, with the limit of detection at 5 µg/kg and 0.25 µg/kg, respectively. Accuracy ranged from 106–119%. Precision was below 7% the relative standard deviation for both analytes. The method was successfully applied for the determination of trimethylamine-*N*-oxide and trimethylamine contents in nine commercially available liquid fish oils and three commercially available fish oil capsules, showing that trimethylamine and trimethylamine-*N*-oxide are not present in highly refined fish oils.

## 1. Introduction

According to the World Health Organization, cardiovascular diseases (CVD) are the leading cause of death globally, taking an estimated 17.9 million lives each year [1]. One popular way to reduce risk for CVD is supplementing fish oil, as several studies have shown a correlation between fish oil consumption and a reduced risk of suffering from CVD [2,3]. Hence, according to the United States National Institute of Health, fish oil is one of the most common non-vitamin and non-mineral dietary supplements used by Americans [4,5]. The main beneficial compounds in fish oil are omega-3 polyunsaturated fatty acids, shaping the membranes of the cardiovascular system [2,3]. However, especially fish oils from marine fish can also contain undesirable compounds such as trimethylamine (TMA) and trimethylamine-*N*-oxide (TMAO), as well as further biogenic amine derivatives. TMAO is a zwitterionic organic molecule that recently emerged as a candidate risk factor for CVDs, being associated with atherosclerosis, heart failure, hypertension, and other CVDs [6,7]. It is introduced into the human bloodstream either by direct consumption or through conversion of TMA by hepatic flavin monooxygenases [8,9]. While one source of TMA is the transformation of choline and carnitine by the human gut microbiota in the large intestine, readily available TMA from food is also absorbed by intestinal cells and quickly enters the portal blood for subsequent conversion to TMAO in the liver [9]. Furthermore, TMAO from food has also been shown to be absorbed in the gut and to quickly enter the bloodstream [10]. In mammalian kidneys, TMAO is accumulated to counteract the destabilization of macromolecular structures like proteins and nucleic acids by urea, as well as to offset its inhibitory effects on functions such as ligand binding [11]. In marine organisms, TMAO acts as an organic osmolyte for maintaining cell volume [11]. After the harvest of fish and shellfish, bacteria chemically reduce TMAO, consequently increasing the levels of TMA [12]. Hence, fish, shellfish, and the products thereof are the main dietary sources of TMAO and TMA, with concentrations of TMAO from below 5 mg/kg up to 3200 mg/kg and concentrations for TMA ranging from below 2 mg/kg up to 550 mg/kg being reported for the main traded fish species in Hong Kong [13].

Comprehensive analysis of TMAO and its precursors such as TMA in foodstuffs is required for exposure estimations in humans. However, TMAO and TMA, although structurally quite similar, possess different characteristics relevant for their analysis, especially regarding their volatility. TMA is a highly volatile (boiling point: 3 °C) organic amine. Therefore, it is primarily analyzed by headspace gas chromatography (HS-GC) in combination with mass spectrometry (MS) [14,15]. TMAO, on the other hand, is non-volatile and, therefore, rarely analyzed by GC-MS. The most popular method for their simultaneous determination in foodstuffs is capillary electrophoresis, as proposed for fish extracts by Timm and Jorgensen [16]. This method is fast, but requires expensive specialized equipment, and reproducible detector responses are hard to obtain [17]. Thus, a new method for the determination of dimethylamine, TMA, and TMAO in aquatic products, using ion chromatography with non-suppressed conductivity detection after extraction with trichloroacetic acid, was proposed by Li et al. [17]. However, the required equipment is not necessarily less specialized, although being less expensive. Hefni et al. proposed hydrophilic interaction liquid chromatography (HILIC) with mass spectrometry (MS) detection for the simultaneous quantification of TMA and TMAO along with other methylamines in clinical and food samples [18]. This method was applied to eggs and meatballs and has the inherent disadvantage of requiring a laborious derivatization step for TMA detection, which increases the analysis time and the use of resources and makes this method more prone to errors. Lastly, an ultra-high performance liquid chromatography (UHPLC)-MS method was applied for the quantification of TMA and TMAO in hydrogen peroxide-altered fish samples [19].

While plenty of methods are discussed for the determination of TMA and TMAO in fish, there does not yet exist a method for the quantification of TMA and TMAO in significantly lipophilic matrices. However, similar disadvantages as described for the other methods need to be also overcome for lipophilic matrices. Additionally, extraction from significantly lipophilic matrices may require different extraction conditions and solvents from the ones proposed in the studies published [17,18,19]. Furthermore, the analysis of TMA and TMAO is of importance to estimate their impact in fish oil supplements on circulating levels of TMAO in the bloodstream and, thus, potential adverse effects on human health.

Consequently, the present study aimed at developing and validating a quick and simple derivatization-free liquid–liquid extraction method followed by hydrophilic interaction liquid chromatography tandem-MS (HILIC-MS/MS) separation for analyzing TMA and TMAO in fish oils (and supplements, i.e., capsules, containing these). To obtain an overview of the quality and range of concentrations, nine commercially available liquid fish oils and three commercially available fish oil capsules were exemplarily analyzed for their TMA and TMAO contents.

## 2. Results and Discussion

The results obtained were primarily planned and are discussed regarding the method’s aptitude for quick and easy derivatization-free analysis of TMA and TMAO in fish oils for human consumption.

### 2.1. Detection Method Optimization

Detection for all analytes was optimal in positive ion mode as the responses were higher than in negative ion mode. The precursor ions, product ions, the quantifier and qualifier, and the optimal CE and CAV are given in Table 1. The dwell time was set to 10 ms for all transitions. Retention times were 0.9 min for triethylamine (TEA), 1.2 min for TMA, and 1.2 min for TMAO. Hence, the liquid chromatography tandem-mass spectrometry (LC-MS/MS) method can even be reduced to as little as three minutes, if a higher sample throughput is required. However, doing so would decrease the column cleanup during the run and, thereby, possibly the lifetime of the column itself. Despite co-elution, MS/MS detection enabled selective detection and, thus, quantification of TMA and TMAO.

### 2.2. Method Development

The recovery rates for TMAO for all three extraction solvents ranged from 94 to 99%. For TMA, eluent A and 1% formic acid in methanol resulted in 89% and 96% recovery rates, respectively. The recovery rate for samples in 0.5 M trichloroacetic acid was 336%. As the recovery rates were considered acceptable within a range of 70–120%, according to U.S. Food and Drug Administration (FDA) guidance [20], trichloroacetic acid was excluded from further consideration. Furthermore, regarding peak symmetries, the use of eluent A resulted in a very good symmetry, while the use of methanol with 1% formic acid showed a prevalence for peak shoulder formation. Additionally, unlike with 1% formic acid in methanol, extraction with eluent A did not require the preparation of an additional solution, as eluent A needs to be prepared for the subsequent HILIC-MS/MS analysis. Thus, extraction with eluent A showed better peak symmetries and an overall reduced analysis time, rendering it the most advantageous extraction solvent of the three ones tested. In the next step, the sample cleanup was investigated. Therefore, two samples were extracted with eluent A in duplicate. One of each duplicate sample was subsequently extracted with the same amount of n-hexane by thoroughly mixing. Afterwards, 1 mL of the eluent A phases of each sample was transferred to a 1.5 mL glass vial and dried in an oven. All samples were then weighed, and the net weight of the residue was calculated by subtracting the tare of the glass vials. The average weight of the residue without additional n-hexane extraction was 1.08 ± 0.09 mg/mL. The average weight of the residue after additional extraction with n-hexane was 1.04 ± 0.13 mg/mL. The difference in these values was below the measurement certainty of the scale applied. Thus, no significant improvement in sample cleanup resulted from a second extraction step with n-hexane. Hence, this step was not adopted for the final method. Lastly, the necessity of conducting a matrix calibration was investigated by calculating the recovery rates of a matrix calibration measured against an external calibration with an internal standard. Except for the 2 ng/mL level for TMAO, all matrix calibration levels yielded recovery rates from 88–105% for both analytes in both blank samples. The mean values were 96% and 95% for TMA with standard deviations of 6% and 4%, respectively, as well as 89% and 102% for TMAO with standard deviations of 8% and 2%, respectively. Hence, all results were well within the ranges of 70–120% accuracy and <15% precision, as given by the FDA guidelines. Therefore, the external calibration was considered equivalent to the matrix calibration. The application of an external calibration further reduced the time of work required and, thus, the overall analysis time. In conclusion, eluent A showed the best extraction properties, considering extraction efficiency, peak symmetry, and overall handling. Also, further extraction with n-hexane did not result in a significant improvement of the sample cleanup, indicating that eluent A did not extract notable amounts of lipophilic compounds. Furthermore, external calibration with an internal standard was shown to be equivalent to matrix calibration.

### 2.3. Method Validation

**Figure 1:**
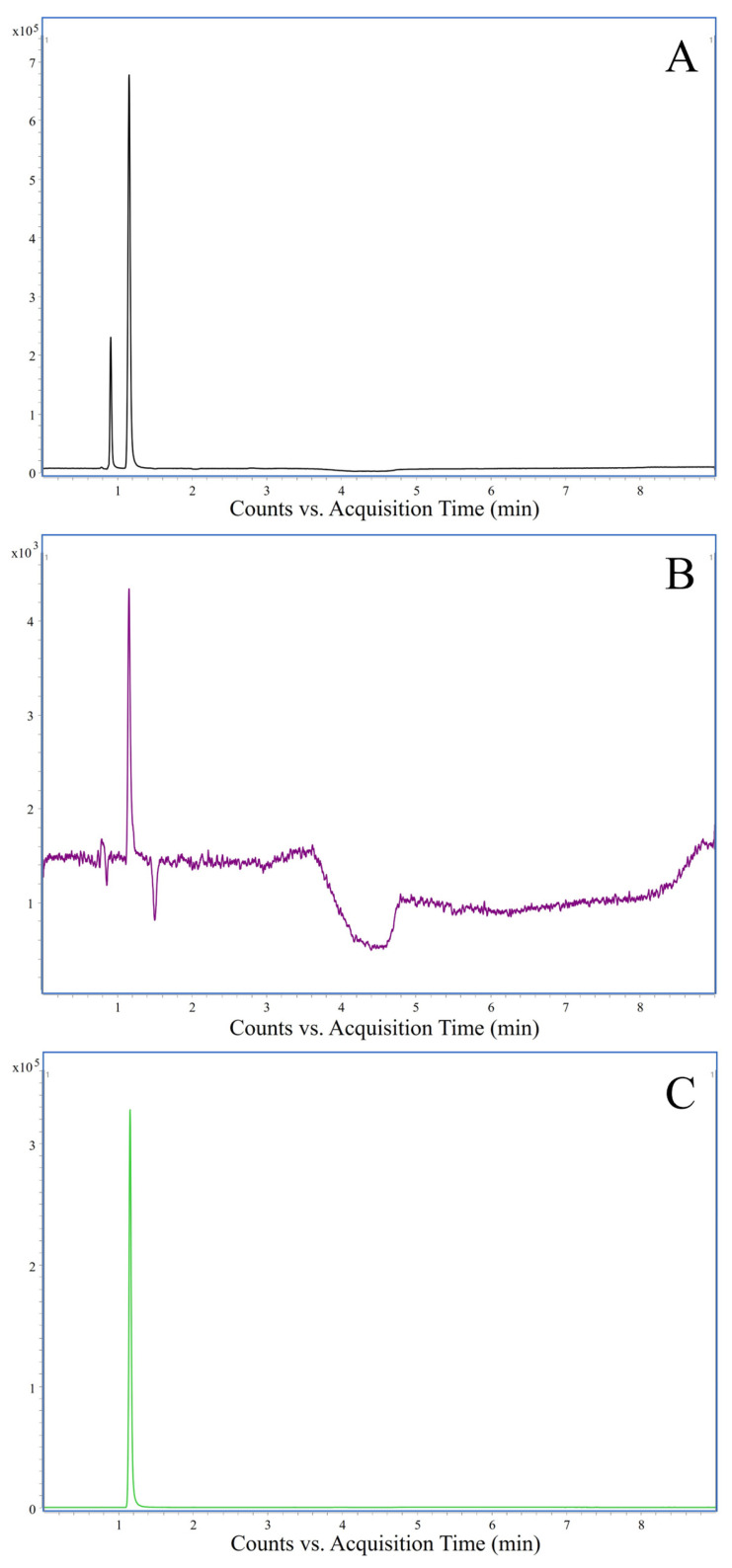
Chromatograms of a standard mixture at the LLOQ. (**A**) Total ion chromatogram; (**B**) chromatogram of the quantifier transition of TMA; (**C**) chromatogram of the quantifier transition of TMAO.

Validation data for TMA and TMAO are shown in Table 2, Table 3 and Table 4. Linearity was confirmed in the ranges of 100–1000 ng/mL for TMA and 10–100 ng/mL for TMAO. The coefficients of determination (R^2^) for both compounds were higher than 0.995 and are given in Table 2 along with the slope and intercept of the acquired calibration curves. Also, the lack-of-fit test and visual inspection of residual plots passed. Thus, a high linearity was proven. The lowest limit of quantification (LLOQ) values were 100 µg/kg for TMA and 10 µg/kg for TMAO, while the limit of detection (LOD) and limit of quantification (LOQ) values were 5 µg/kg and 10 µg/kg for TMA, as well as 0.25 µg/kg and 0.75 µg/kg for TMAO, respectively (Table 3). Chromatograms of a standard mix at the LLOQ are shown in Figure 1.

In comparison with the capillary electrophoresis method, as proposed by Timm and Jørgensen [16], this method showed increased sensitivity by lowering the linear range from approximately 1.5 µg/mL–150 µg/mL for both analytes to 10–100 ng/mL for TMAO and 100–1000 ng/mL for TMA, the LOD from ~1 mg/L for both analytes to 0.25 µg/kg for TMAO and 5 µg/kg for TMA, and the LOQ from approximately 3 mg/L for both analytes to 0.75 µg/kg for TMAO and 10 µg/kg for TMA. Similarly, this method showed a superior LOD compared to the ion exchange chromatography method proposed by Li et al. [17], lowering the LOD of TMA from 0.08 mg/L to 5 µg/kg and the LOD of TMAO from 0.10 mg/L to 0.25 µg/kg. Hefni et al. reported the LOD for TMA and TMAO to be 2.9 ng/mL and 4.13 ng/mL, respectively [18]. However, the reported value refers to the extract only. When calculating the LOD in relation to the employed food source, LOD values of 116 µg/kg for TMA and 165 µg/kg for TMAO result. Accuracy for the presented method was between 113 and 119% for TMA with intraday precision ranging from 1–3% and inter-day precision at 4–7% (Table 4). The determination of TMAO showed an accuracy of 106–113% with intra-day and inter-day precisions between 1–3% and 3–6%, respectively (Table 4). The precision results were within the acceptable range of ± 15% standard deviation, as well as ± 20% standard deviation for the LLOQ, as recommended by the FDA guideline. The obtained values for precision were in line with the previously reported values by Timm and Jørgensen [16], Li et al. [17], Hefni et al. [18], and Dal Bello et al. [19], who obtained precision values ranging from approximately 3 to 11% for both compounds. However, accuracy was only reported by Dal Bello et al. [19] and Hefni et al. [18] in the form of recovery and compared well to the results obtained with the newly developed method, giving values ranging from 75.8 to 102.5% for TMAO and 86.0 to 97.9% for TMA [17,18]. In the present study, carry-over was found to be below 1% for both analytes, as well as the internal standard and, thus, below the acceptable threshold of 20% of the response at the LLOQ for the analytes and 5% for the internal standard. Hence, the developed method showed good accuracy and precision, as well as high sensitivity and linearity with little carry-over. The method validation showed that the presented method is well-suited for the analysis of TMA and TMAO in oils for human consumption also in comparison with methods for other matrices published before.

### 2.4. Analysis of Commercially Available Fish Oils

**Figure 2:**
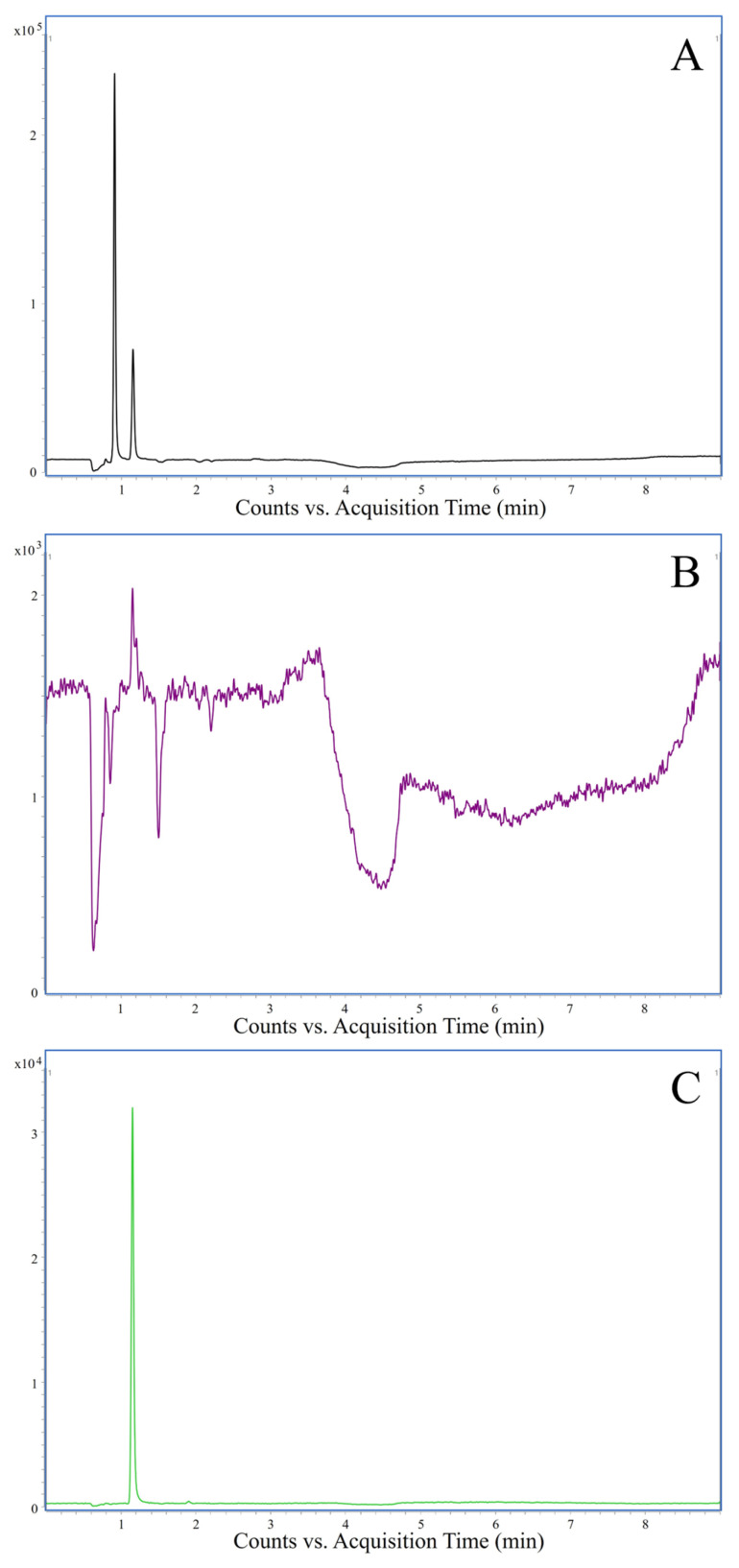
Chromatograms of a fish oil sample with analyte concentrations below the LOQ. (**A**) Total ion chromatogram; (**B**) chromatogram of the quantifier transition of TMA; (**C**) chromatogram of the quantifier transition of TMAO.

Nine liquid fish oil samples and three fish oil capsules were investigated exemplarily for their TMA and TMAO contents to have an impression of the range and variety present in commercially available fish oils. The results are shown in Table 5. TMA and TMAO were not detectable in any of the liquid fish oil samples. Similar results were found for the fish oil capsules, as TMA and TMAO were found to be below the LLOQ in all three samples. However, TMA was found in two of the samples with contents higher than the LOD, but lower than the LLOQ. TMAO was detectable above the LOQ and below the LLOQ in one sample. Exemplary chromatograms of the results of one fish oil with TMA and TMAO contents above the LOD are shown in Figure 2. All samples were spiked with TMA and TMAO at the LLOQ level, and the resulting recoveries ranged from 81 to 104% for TMA and 86 to 110% for TMAO (Table 5). Overall, no significant amounts of TMA and TMAO were found in any of the fish oils analyzed in the present study, even though high concentrations of up to 2200 mg TMAO/kg fish and 99 mg TMA/kg fish have been reported for cod [13]. This is most likely due to the processing applied during fish oil production. TMA and TMAO possess a strong fishy odor [21]. Hence, they are most likely removed during processing to ensure sensory acceptance by the customer. Off-flavor attributes like the ones originating from TMA and TMAO are still a concern for many customers, as they result in so-called “fish burps” [22]. Originally, these off-flavors were masked by encapsulating the oils [23]. Besides fish oil supplementation, encapsulation aims also at providing fish oils as ingredients for the fortification of products such as yoghurt, goat cheese, and other products [24,25,26]. Consequently, refining processes are necessary [27]. Usually, one of the processing steps is deodorization, where odorous compounds are removed by steam distillation or other means, although researchers are working on gentler options to remove odors, which require less energy than high-temperature steam distillation [27]. However, residual TMA and TMAO might be present, as the removal rates of some of the processing methods are significantly lower [27]. Furthermore, the refinement process is rarely complete, as sensory evaluation of fish oil still shows a fishy odor even after deodorization due to the incomplete deodorization. This results in volatile compounds still being present in the marketed products [28]. A further important fact to consider is that the raw materials used for fish oil production originate from a plethora of sources. While cod liver oil is the prevalent source for fish oil in Germany, in other regions of the world, fish oil is also produced from different species [29]. Also, whereas the German consumer market does not embrace strong fishy flavors, other countries indulge in delicacies like surströmming (Swedish sour herring) or Icelandic hakarl (fermented or cured shark), which are famous for their overpowering fishy odor [30].

## 3. Materials and Methods

### 3.1. Chemicals

TEA hydrochloride (98%) and TMAO (95%) were purchased from Sigma-Aldrich Chemie GmbH (Schnelldorf, Germany). TMA hydrochloride (for synthesis) was acquired from Merck KGaA (Darmstadt, Germany). Trichloroacetic acid (100%), formic acid (98%), acetonitrile (MeCN), and water of LC-MS grade were obtained from VWR International GmbH (Darmstadt, Germany). Ammonium formate (>99%, for LC-MS) was acquired from Carl Roth GmbH & Co. KG (Karlsruhe, Germany). Hydrochloric acid (>37%) was obtained from Honeywell Deutschland Holding GmbH (Offenbach, Germany). Methanol (>99.95%) and hexane (>95%) were purchased from Th. Geyer GmbH & Co. KG (Renningen, Germany). Syringe filters Chromafil Xtra RC-20/25 were acquired from Macherey-Nagel GmbH & Co. KG (Düren, Germany).

### 3.2. Sample Material

Organic olive (*Olea europaea*) oil and fish oil capsules were acquired from a local supermarket. Blank fish oil samples for method development, i.e., fish oil where TMA and TMAO have been removed through processing steps, were provided by NORSAN GmbH (Berlin, Germany). A total of nine fish oils of different origins for quantification of TMA and TMAO were purchased online. Two of these samples were marketed for children (as mentioned on the declaration). All nine liquid fish oil samples were cod or cod liver oil (as mentioned on the declaration), primarily. One of the samples catering to children also contained around 20% olive oil (as mentioned on the declaration). No flavoring was added to three liquid fish oil samples, while four samples contained lemon flavoring and two samples contained orange flavoring. Vitamin E was added to all samples, with vitamin D3 added to five samples and vitamin A added to one sample. The fish oil capsules contained sea fish oil with one sample also declaring <5% salmon oil. Vitamin E was also added to all fish oil capsules. The number and type of fish oil samples were chosen to represent the easily obtainable fish oil supplements on the market.

### 3.3. Sample Preparation

Samples were stored in a refrigerator at 4–8 °C until use. Next, 5 ± 0.05 g of sample material was weighed into 15 mL screw cap tubes, and 5 mL of extraction solvent was added. The mixture was then shaken vigorously for 5 min before centrifugation (3494× *g*, 5 min). The supernatant was membrane-filtered before injection into the UHPLC electrospray ionization (ESI) tandem-MS (MS/MS) system.

### 3.4. UHPLC-ESI-MS/MS Analysis

UHPLC-ESI-MS/MS was performed on an Agilent Technologies 1260 Infinity II LC system equipped with an Agilent Technologies 6495C triple quadrupole mass spectrometer (Agilent Technologies Deutschland GmbH, Waldbronn, Germany). After extensive method development, the amines were separated on a Poroshell 120 HILIC-Z column (150 mm × 2.1 mm, 2.7 µm) in combination with a Poroshell 120 HILIC-Z guard column (5 mm × 2.1 mm, 2.7 µm) obtained from Agilent Technologies Deutschland GmbH (Waldbronn, Germany). The column was heated to 40 °C in a column heater. The mobile phase consisted of two eluents: 90:10 (*v*/*v*) ACN–water (A) and water (B) to both of which 50 mM ammonium formate and 2.5% formic acid were added. The elution program was started with 100% eluent A and held for 2 min. The percentage of eluent B was then linearly raised to 50% over 3 min. Within 2 min, the percentage of eluent B was reduced to 0%, and the column was then equilibrated for another 2 min. The mobile phase flow was set constant at 0.5 mL/min. The injection volume was set to 1 µL. UHPLC-ESI-MS/MS data were acquired and processed by the use of the MassHunter QQQ Quantitative Analysis software version B.08.00 (Agilent Technologies Deutschland GmbH, Waldbronn, Germany).

### 3.5. Stock Solutions, Calibration Standards, and Quality Control Sample Preparation

Next, 10 g/L stock solutions in 1 M hydrochloric acid were prepared for each analyte and stored at −20 °C for a maximum of four weeks. Olive oil was used as a quality control sample, because it does not naturally contain TMA or TMAO. Calibration standard solutions were made in a range from 10 ng/mL to 100 ng/mL for TMAO and from 100 ng/mL to 1000 ng/mL for TMA. TEA was added as an internal standard to each calibration solution to reach a final concentration of 50 ng/mL. For accuracy and precision measurements, blank samples were spiked with standard solution in eluent A. Concentration levels for spiked samples were 50 ng, 100 ng, 250 ng, and 400 ng for TMAO and 500 ng, 1000 ng, 2500 ng, and 4000 ng for TMA. Then, 250 ng of TEA was added to every sample as the internal standard. All standards were prepared freshly every day from the stock solutions.

### 3.6. Detection Method Optimization

For optimization of the ESI-MS/MS detection of the analytes, 1 µL of 100 ng/mL standard solutions was injected directly into the MS/MS system for each analyte and step. Firstly, the optimal polarity was determined by performing a full scan in positive and negative ion mode and the comparison of the resulting peaks in the spectra, which overlapped with the calculated masses. Then, a product ion scan was conducted to look for the most intense product ions by comparing mass spectral intensity. Subsequently, the collision energy (CE) and cell accelerator voltage (CAV) were optimized by alternating the values in multiple-reaction-monitoring (MRM) mode and selecting the values resulting in high peak areas and signal-to-noise ratios (S/N).

### 3.7. Extraction Method Development

Three extraction solvents (0.5 M trichloroacetic acid, eluent A, and 1% formic acid in methanol) were investigated for the best extraction efficiency. Trichloroacetic acid (TCA, 0.5 M) was chosen, as it is often used for amine extraction from fish samples [31]. Formic acid 1% is traditionally used for other analytes containing amine groups and was chosen to reduce the extraction solvent’s elution strength. The latter is also the reason for using eluent A as an extraction solvent, as 100% eluent A is the starting condition for the liquid chromatographic method. All three extraction solvents created a two-phase system after addition to the fish oil. For every extraction solvent, two samples of a salmon oil sample with a fishy odor, indicative of the presence of TMA, were weighed into screw cap tubes. For each extraction solvent, one sample was spiked. The samples extracted with eluent A and 1% formic acid in methanol were spiked with 2500 ng of TMA and 250 ng of TMAO, while the sample to be extracted with trichloroacetic acid was spiked with 25,000 ng of TMA and 2500 ng of TMAO. The higher concentration for samples extracted with trichloroacetic acid is necessary due to peak splitting being prevalent in purely aqueous solutions, requiring a 1:10 dilution step before injection. Hence, TCA as the extraction solvent would result in higher limits of detection and quantification than 1% formic acid in methanol and eluent A. All samples were measured against an external calibration, consisting of 10 equidistant points ranging from 10 to 100 ng/mL for TMAO and 100 to 1000 ng/mL for TMA. To evaluate the extraction efficiency of the extraction solvents, the difference between the measured analyte content of the spiked and non-spiked samples was divided by the known amount of analyte spiked. The peak symmetries and overall handling of the different extraction solvents were also taken into consideration. After the best-suited extraction solvent was determined, sample cleanup was investigated. Therefore, 2 mL of the extract obtained with the best extraction solvent was subsequently extracted with 2 mL of n-hexane to extract lipophilic compounds. Then, 1 mL of the extract gained after extraction with n-hexane was placed into a 1.5 mL glass vial and dried in an oven at 70 °C. The same was performed with the extract obtained before extraction with n-hexane. Both samples were analyzed gravimetrically (*n* = 2). Lastly, it was investigated whether a calibration by standard addition is required or if an external calibration with the use of an internal standard would suffice. For this, 20 g of two different blank sample materials was extracted with 20 mL extraction solvent each, and the extracts were spiked with five equidistantly chosen amounts of the standard, resulting in concentrations ranging from 2 to 10 ng/mL for TMAO and from 100 to 500 ng/mL for TMA. These samples were then measured against a calibration consisting of eluent A spiked with standards resulting in the same concentration ranges. This experiment was evaluated by calculating the measured amounts and dividing them by the known amounts of added analyte.

### 3.8. Method Validation

The analysis of TMAO and TMA in different edible oils was validated to obtain the calibration model, accuracy and precision, LOD. and LOQ, as well as carry-over according to the guidance of bioanalytical method validation published by the FDA with modifications where necessary [19]. The linearity of the method was confirmed by the analysis of a ten-point standard curve (*n* = 3) ranging from 10 ng/mL to 100 ng/mL for TMAO and from 100 ng/mL to 1000 ng/mL for TMA. The accuracy and precision values were determined fivefold at every concentration level. Precision was determined intra-day (*n* = 5) and inter-day (*n* = 5), with inter-day samples being analyzed on three different days by three different coworkers of the lab. The LOD and LOQ were determined by spiking blank samples with multiple low analyte concentrations. The LOD and LOQ for TMA were defined as the concentration resulting in a S/N of 3 and 10, respectively. This was not possible for TMAO, as a very small, but constant peak was detected in every sample. Thus, the LOD and LOQ for TMAO were defined as the lowest value to exceed the blank peak area by factors of 3 and 10, respectively. Carry-over was determined by injecting a solvent blank after the highest calibration level and dividing the resulting peak areas by the areas of the LLOQ.

### 3.9. Analysis of Commercially Available Fish Oils

Liquid fish oils and fish oil capsules were stored at 4–8 °C until processing. Liquid fish oils were vigorously shaken and transferred into screw cap tubes for analysis. Fish oil capsules were cut open, and their contents were squeezed into screw cap tubes. Then, 5 ± 0.05 g of each sample was weighed into the screw cap tubes twice. For each sample, one aliquot was spiked with 50 ng of TMAO, 500 ng of TMA, and 250 ng of TEA.

## 4. Conclusions

A reliable and highly sensitive HILIC-ESI-MS/MS method for the quantification of TMA and TMAO without derivatization and with a quick and easy sample preparation was developed. This method shows an improved sensitivity compared to the capillary electrophoresis method proposed by Timm and Jørgensen, as well as the ion chromatography with suppressed conductivity detection method proposed by Li et al. [16,17]. The capillary electrophoresis method is more time consuming and cannot be run unattended over a long period of time, for example overnight. Thus, its use in service laboratories is less economical than the presented method. Additionally, the developed method requires less time and resources than the method proposed by Hefni et al. [18], as it does not need a derivatization step and does not employ standard addition for quantification of TMA and TMAO. Furthermore, by employing LC with MS/MS detection, no additional specialized equipment is required. Also, this method is the only one tailored specifically to be used with significantly lipophilic matrices such as oils for human consumption and fish oils in particular. Thus, this method is well suited for the analysis of TMA and TMAO in oils, also with regard to routine analysis in service laboratories. Also, when an even higher sample throughput is required, the method length may be reduced from nine to as little as three minutes, thus saving even more time and resources. Hence, this method will enable quick and reliable detection of TMA and TMAO.

The applicability of this method for TMA and TMAO exposure estimation has been further proven exemplarily by analysis of fish oil samples. The analysis of nine commercially available liquid fish oils did not detect any TMA or TMAO. The analysis of three commercially available fish oil capsules resulted in values of less than 100 µg/kg TMA and less than 10 µg/kg TMAO. Hence, commercially available fish oil, when processed to the newest technological standards, is unlikely to contribute to the circulating TMA and TMAO in human blood. Nonetheless, the presence of residual TMA and TMAO in some of the present samples showed that regular analysis of these compounds as a part of the quality control process may be reasonable to reduce the risk of adverse health effects for consumers. It will be helpful for exposure estimation and presents as a reasonable parameter to control as part of the manufacturer’s quality assurance process. However, no regulation limiting the concentrations of TMA and TMAO in foodstuffs has been established to this date.

## Figures and Tables

**Table 1:** Optimized MS/MS parameters for TMA, TMAO, and TEA.

Compound	Precursor Ion [*m*/*z*]	Quantifier Ion [*m*/*z*]	Qualifier Ion [*m*/*z*]	Quantifier CE [V]	Quantifier CAV [V]
TEA	102	58	74	24	8
TMA	60	44	28	24	8
TMAO	76	58	59	25	5

*m*/*z*: mass-to-charge ratio, CE: collision energy, CAV: cell accelerator voltage, TEA: triethylamine, TMA: trimethylamine, TMAO: trimethylamine-*N*-oxide.

**Table 2:** Validation results for HILIC-ESI-MS/MS analysis of TMA and TMAO in oil.

Compound	R^2^	Linear Range [ng/mL]	Slope	Intercept
TMA	0.9984	100–1000	0.039	0.002
TMAO	0.9986	10–100	31.915	0.253

R^2^: coefficient of determination, TMA: trimethylamine, TMAO: trimethylamine-*N*-oxide.

**Table 3:** Validation data for HILIC-ESI-MS/MS analysis of TMA and TMAO in oil.

Compound	LOD [µg/kg]	LOQ [µg/kg]	LLOQ [µg/kg]	Carry-Over [%]
TMA	5	10	100	0.41
TMAO	0.25	0.75	10	0.70

LOD: limit of detection, LOQ: limit of quantification, LLOQ: lowest limit of quantification (the lowest concentration of analytes, which can be quantitatively measured with suitable accuracy and precision), TMA: trimethylamine, TMAO: trimethylamine-*N*-oxide.

**Table 4:** Validation data for HILIC-ESI-MS/MS analysis of TMA and TMAO in oil.

Compound	Accuracy [%]				Intra-Day Precision RSD [%]				Inter-Day Precision RSD [%]			
	Q	L	M	H	Q	L	M	H	Q	L	M	H
TMA	113	115	117	119	2	3	1	1	7	4	4	5
TMAO	106	109	113	113	3	2	1	1	6	4	3	3

Blank oil samples were spiked at four different levels of 500 ng (Q), 1000 ng (L), 2500 ng (M), and 4000 ng (H) of TMA, as well as 50 ng (Q), 100 ng (L), 250 ng (M), and 400 ng (H) of TMAO (*n* = 5). RSD: relative standard deviation, TMA: trimethylamine, TMAO: trimethylamine-*N*-oxide.

**Table 5:** Results of TMA and TMAO quantification in nine liquid fish oil samples and three fish oil capsules.

Sample	TMA Content [µg/kg]	Recovery Rate [%]	TMAO Content [µg/kg]	Recovery Rate [%]
Fish oil liquid 1	n.d.	97	n.d.	96
Fish oil liquid 2	n.d.	81	n.d.	96
Fish oil liquid 3	n.d.	104	n.d.	96
Fish oil liquid 4	n.d.	101	n.d.	94
Fish oil liquid 5	n.d.	103	n.d.	99
Fish oil liquid 6	n.d.	101	n.d.	97
Fish oil liquid 7	n.d.	104	n.d.	95
Fish oil liquid 8	n.d.	104	n.d.	101
Fish oil liquid 9	n.d.	95	n.d.	86
Fish oil capsule 1	n.d.	100	n.d.	109
Fish oil capsule 2	16 *	102	n.d.	110
Fish oil capsule 3	23 *	102	1 *	107

Fish oil samples were spiked with 500 ng of TMA and 50 ng of TMAO. * Concentrations are below the lowest limit of quantification (LLOQ), but higher than the absolute limit of quantification (LOQ). TMA: trimethylamine, TMAO: trimethylamine-*N*-oxide, n.d.: not detectable.

## Data Availability

The raw data supporting the conclusions of this article will be made available by the authors on request.
